# The Role of Phosphoinositide 3-Kinases in Neutrophil Migration in 3D Collagen Gels

**DOI:** 10.1371/journal.pone.0116250

**Published:** 2015-02-06

**Authors:** Kayleigh J. S. Martin, Michelle J. Muessel, Christine E. Pullar, Gary B. Willars, Andrew J. Wardlaw

**Affiliations:** 1 Institute for Lung Health, Department of Infection, Immunity and Inflammation, University of Leicester, Leicester, United Kingdom; 2 Department of Cell Physiology and Pharmacology, University of Leicester, Leicester, United Kingdom; University Hospital Medical Centre, GERMANY

## Abstract

The entry of neutrophils into tissue has been well characterised; however the fate of these cells once inside the tissue microenvironment is not fully understood. A variety of signal transduction pathways including those involving class I PI3 Kinases have been suggested to be involved in neutrophil migration. This study aims to determine the involvement of PI3 Kinases in chemokinetic and chemotactic neutrophil migration in response to CXCL8 and GM-CSF in a three-dimensional collagen gel, as a model of tissue. Using a three-dimensional collagen assay chemokinetic and chemotactic migration induced by CXCL8 was inhibited with the pan PI3 Kinase inhibitor wortmannin. Analysis of the specific Class I PI3 Kinase catalytic isoforms alpha, delta and gamma using the inhibitors PIK-75, PIK-294 and AS-605240 respectively indicated differential roles in CXCL8-induced neutrophil migration. PIK-294 inhibited both chemokinetic and chemotactic CXCL8-induced migration. AS-605240 markedly reduced CXCL8 induced chemokinetic migration but had no effect on CXCL8 induced chemotactic migration. In contrast PIK-75 inhibited chemotactic migration but not chemokinetic migration. At optimal concentrations of GM-CSF the inhibitors had no effect on the percentage of neutrophil migration in comparison to the control however at suboptimal concentrations wortmannin, AS-605240 and PIK-294 inhibited chemokinesis. This study suggests that PI3 Kinase is necessary for CXCL8 induced migration in a 3D tissue environment but that chemokinetic and chemotactic migration may be controlled by different isoforms with gamma shown to be important in chemokinesis and alpha important in chemotaxis. Neutrophil migration in response to suboptimal concentrations of GM-CSF is dependent on PI3 Kinase, particularly the gamma and delta catalytic isoforms.

## Introduction

Neutrophil accumulation in the tissue plays an important role in host defence to a wide range of infections. However, in chronic inflammatory diseases neutrophil accumulation within tissue can be detrimental. The entry of neutrophils into the tissue has been well characterised [1–3], however, the fate of these cells once inside the tissue microenvironment is not fully understood. An understanding of the signal transduction pathways controlling the migration of neutrophils within the lung could prove beneficial in the treatment of inflammatory diseases.

There are three main forms of leucocyte migration, random, chemokinesis and chemotaxis. Both random and chemokinesis are non-directional motions the difference being that chemokinesis occurs in the presence of a (usually chemical), stimuli and random motion occurs in the absence of such stimuli. Chemotaxis is induced in response to a chemical stimulus, usually a chemoattractant but unlike chemokinesis the migration is directed towards the source of the stimulus [4].

PI3Ks have been suggested to play an important role in neutrophil migration, with many authors showing substantial inhibition in the absence of PI3K [5–7]. Activation of receptor tyrosine kinases or G-protein coupled receptors lead to the activation of class I PI3Ks, which are responsible for controlling the phosphorylation of phosphatidylinositol (4,5)-bisphophate to form phosphatidylinositol (3,4,5)-trisphosphate [8]. Phosphatidylinositol (3,4,5)-trisphosphate has an important role in the development of cell polarity, which is necessary for cell motility and directional sensing [9, 10].

In the context of neutrophil migration two classes of PI3K are thought to be involved, Class IA and Class IB. Class IA contains the catalytic subunits α, β and δ [11–13]. Class IB contains the catalytic subunit γ [14]. Recent experimental evidence has suggested distinct requirements for the δ and γ subunits in migration and recruitment [14–16]. In response to stimulation with fMLF chemokinesis was shown to be critically dependent on the gamma isoform. However the PI3Kγ was not required for gradient sensing and gradient-biased movement and was therefore not thought to be involved in chemotaxis [17]. In contrast fMLF-induced chemotaxis was dependent on the delta isoform whereas chemokinesis was PI3Kδ independent [15]. In addition PI3Kγ was vital for early neutrophil migration into inflamed tissue whereas in delayed neutrophil emigration in response to neutrophil chemokines, PI3Kδ replaces PI3Kγ and maintains the migration [16].

The Boyden chamber assay is used in the majority of the literature to study neutrophil migration. However, this assay is essentially two-dimensional (2D) and does not relate closely to the environment encountered by the neutrophil once it enters tissue, which presents migrational clues in a 3D context with signals from the tissue matrix and tissue resident cells and structures. There may be significant differences in the adhesive, chemoattractant and signalling processes between 2D and 3D contexts. For example, leukocyte migration over a 2D surface is integrin dependent, whereas rapid migration in a 3D matrix can occur in the absence of integrin adhesion, as the cell migrates forward by pushing and squeezing itself between the matrix fibres [18, 19]. Collagen is an important component of the extra-cellular matrix and lung remodelling in asthma is characterized by collagen deposition [19–21]. Leucocytes have been shown to display a similar pattern of migration in 3D collagen gels to that in vivo in peripheral tissue using intravital microscopy [19, 22]. We have therefore used the 3D collagen gel assay to mimic the tissue to enable us to study the involvement of class I PI3Ks in CXCL8- and GM-CSF-induced neutrophil migration. In this study we show that neutrophils migrate in response to stimulation with CXCL8 or GM-CSF. Wortmannin inhibits CXCL8- and GM-CSF- induced neutrophil migration; however the migration was PI3K isoform specific depending on whether the migration was chemokinetic or chemotactic.

## Materials and Methods

### Reagents

GM-CSF and CXCL8 were obtained from R&D Systems. Histopaque was purchased from Sigma-Aldrich. EDTA, Sodium Bicarbonate, 10X MEM, RPMI and HEPES were all obtained from Fischer Scientific. PI3Kδ selective inhibitor (PIK-294) was purchased from Symansis. Wortmannin, PI3Kγ selective inhibitor (AS-605240) and PI3Kα selective inhibitor (PIK-75) were from Calbiochem (Merck). Phospho-Akt and Akt were purchased from Cell Signalling Technology. Collagen I was purchased from Nutacon.

### Neutrophil isolation

Human blood (20mls) was taken from normal volunteers after obtaining written informed consent. The study was approved by the Leicestershire, Northamptonshire and Rutland Research Ethics Committee 2, reference number 09/H0402/4. The red blood cells were sedimented using 6% dextran. Neutrophils were purified by density gradient centrifugation with histopaque. Neutrophils obtained were typically 96% pure.

### Collagen gel and migration

**Fig 1:**
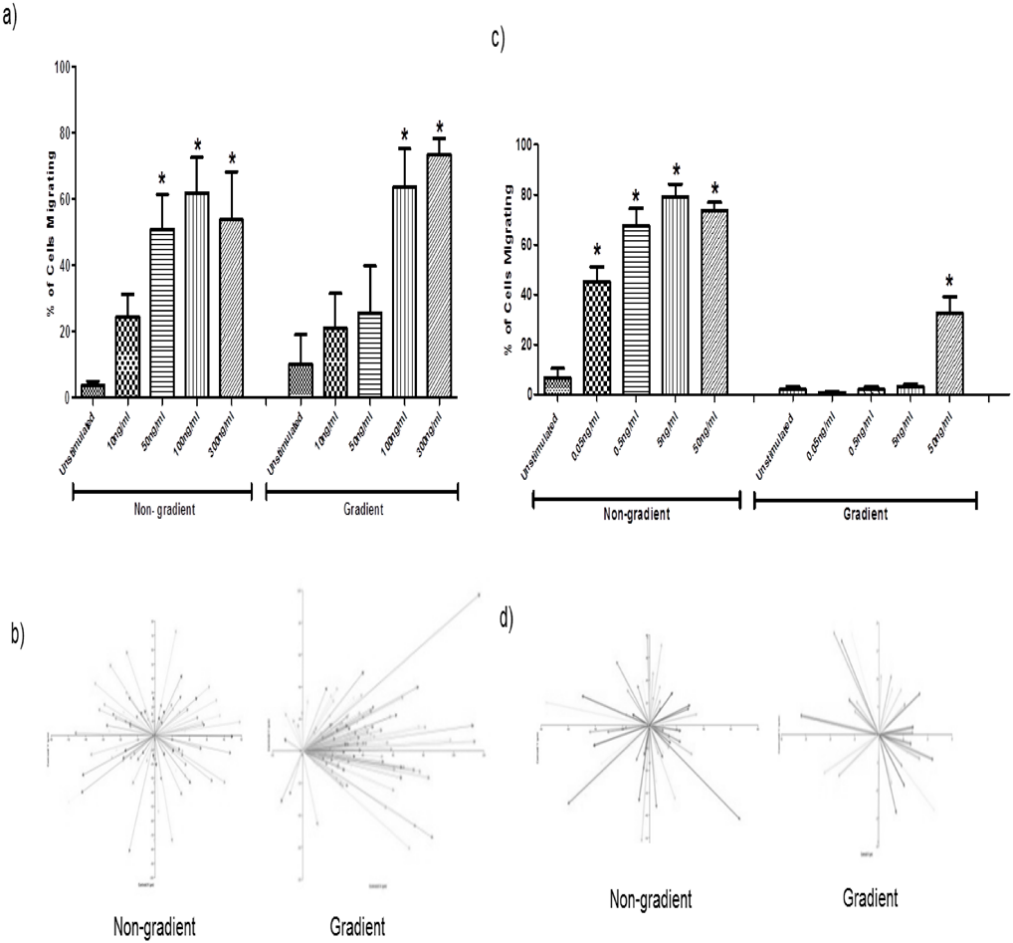
Neutrophil migration is dose dependent in both the gradient and non-gradient assays. a) migration in response to CXCL8, *significantly greater as compared to control cells, p<0.05, n = 3 except for non gradient control and 100ng/ml where n = 5. b) The direction of movement for each neutrophil in the non gradient and gradient assay in response to 100ng/ml of CXCL8 is illustrated in the vector diagrams which show chemokinetic migration in the non-gradient assay and a chemotactic pattern of migration in the gradient assay. c) Neutrophil migration to GM-CSF is dose dependent in both the non gradient and gradients assays. *significantly greater as compared to control cells, p<0.05, n = 3 except for gradient control and 50ng/ml where n = 7 and 6 respectively. d). The direction of movement for each neutrophil in the non gradient and gradient assay in response to 50ng/ml of GM-CSF is illustrated in the vector diagrams, These show that the pattern of migration to GM-CSF was chemokinetic in both the gradient and non-gradient assays.

3D collagen gels were constructed as previously described by Muessel et al [21]. Purified neutrophils (6 x 10^6^ cell/ml) were brought up in 2% FBS and used directly in the migration assays. In all experiments control cells were unstimulated neutrophils. Cells were mixed with collagen I, 10x MEM and sodium bicarbonate. For non gradient migration assays the chemoattractants and GM-CSF were added to the gel, the gel was then loaded into the chamber, which was made from a coverslip sealed with wax on a microscope slide and incubated at 37° for 1 hour prior to videotaping. For gradient migration assays the gel was loaded into approximately 3/4 of the chamber, which was made from a coverslip sealed with wax on a microscope slide and incubated at 37° for 1 hour prior to videotaping. Various chemoattractants were added to the final 1/4. Neutrophil migration was recorded by time-lapse video-microscopy using a Zeiss Axiovert 25 microscope (Germany) with a heated stage at magnification 20x N/A 0.3 and a QImaging Retiga 1300 CCD camera (BC, Canada). The movies were recorded and analysed by commercially available Improvision software (Coventry, UK, Fig. 1). The percentage of migration was calculated as the number of cells that migrated for at least 3 minutes and greater than 10 micrometers from the point of origin.

### Inhibition experiments

Neutrophils at a concentration of 6x10^6^cells/ml were pre-treated with the appropriate concentration of inhibitor or corresponding DMSO vehicle control for 30 minutes prior to the construction of a non-gradient or gradient gel depending on the type of migration being studied. The gels were then constructed and the migration studied as above.

### Western Blotting

Purified neutrophils were solubilised in laemmli buffer (Sigma) and boiled under reducing conditions. Samples were separated by SDS-PAGE (10% (w/v) acrylamide gel) and electrophoretically transferred onto Immobilion P transfer membranes (Millipore). Membranes were blocked in 5% milk (in TBS + 0.1% Tween 20) for 1 hour and then incubated with corresponding primary antibody overnight in 5% BSA. After washing with TBS+ 0.1% Tween20, membranes were subsequently incubated with secondary antibody in blocking solution for 1 hour followed by signal detection. Total Akt was used as a loading control, using the same protocol on stripped membranes.

### RT-PCR

Total RNA was extracted using TRIzol Reagent (Invitrogen) and treated with chloroform, isopropanol and 75% ethanol. Reverse transcription (RT) was carried out using Omniscript RT kit (Qiagen) according to manufacturer’s instructions. Polymerase chain reaction (PCR) was performed using Taq PCR mastermix kit (Qiagen) and the following primers which had been designed using Primer 3, PI3Kα: left primer 5’-AGG GAC CTC AAT TCA CCT CA-3^’^, right primer 5’-ACA TCA AAT TGG GCA TCC TC-3’, PI3Kβ: left primer 5’-AGC GTG GGT AAA TAC GAT GG-3’, right primer 5’- CAG TCT TGT CGC AAA GTC CA-3’, PI3Kδ: left primer 5’-CTG GTG CAG GTG CTC AAG TA-3’, right primer 5’-AAG TGC ATC AGC TCC TTG GT-3’, PI3Kγ: left primer 5’-TTG TGG CCA AAA CAT ACC AA-3’, right primer 5’-AAT CAC AGC GAA CCT CTG CT-3’. Primers were used at a final concentration of 500nM. The PCR cycle was as follows 94° for 3 minutes, 35 cycles of 94° for 30 seconds, 58° for 30 seconds, 72° for 90 seconds followed by a cycle of 72° for 3 minutes. Β-actin was used as a housekeeping control gene. Following PCR samples were run on a 2% agarose gel and visualized under UV light. To confirm the bands corresponded to the genes of interest the DNA was extracted from the gel using the QIAquick gel extraction kit (Qiagen) and the samples were sent for sequencing to the Protein Nucleic Acid Chemistry Laboratory (PNACL) at the University of Leicester.

### Statistics

Results are expressed as means ±SE. All statistics were ANOVA with a Bonferroni post test unless otherwise stated. Significance was achieved at p<0.05. The commercially available software program, GraphPad Prism, was used.

## Results

### Neutrophil migration in 3D collagen gels

To determine the normal migration pattern of neutrophils in a 3D environment, we stimulated the neutrophils with GM-CSF and CXCL8 in a dose-dependent manner. In the absence of stimulation <10% of cells migrated. CXCL8 in the non-gradient assay caused a dose-dependent chemokinetic migration with a maximum of 60% of cells migrating in response to 100ng/ml (Fig. 1a). The average velocity of the neutrophils also increased dose-dependently with 100ng/ml and 300ng/ml stimulation causing the cells to move at 0.16±0.012μm/s and 0.17±0.032μm/s respectively, which was significantly faster compared to unstimulated cells (0.10±0.015μm/s). The chemokinetic migration in response to 100ng/ml of CXCL8 in the non-gradient assay is highlighted in the vector diagram (Fig. 1b), which shows the direction of migration for each neutrophil in one gel. CXCL8 in the gradient assay caused a dose-dependent chemotactic response with a maximum of 75% of cells migrating in response to 300ng/ml (Fig. 1a). Similarly the average velocity also increased in a dose dependent manner with 100ng/ml and 300ng/ml causing a significant increase in velocity to 0.17±0.017μm/s and 0.18±0.009μm/s respectively, compared to unstimulated cells (0.08±0.015μm/s). In this case the vector diagram (Fig. 1b) shows the majority of cells are moving towards the CXCL8 in a chemotactic pattern of migration. GM-CSF induced vigorous migration in the non-gradient assay with a maximum of 79% of cells migrating in response to stimulation with 5ng/ml (Fig. 1c). In addition the average velocity also increased dose dependently, stimulation with 5ng/ml and 50ng/ml caused a significant increase to 0.203±0.009μm/s and 0.20±0.008μm/s respectively, in comparison to unstimulated cells (0.10±0.015μm/s). In contrast only modest migration was seen in the gradient assay, which was only significant at the highest dose of 50ng/ml (Fig. 1c). The average velocity with 50ng/ml was 0.14±0.02μm/s which was significantly higher than unstimulated cells (0.10±0.04μm/s). In contrast to CXCL8 both the gradient and non-gradient assays resulted in chemokinetic patterns of stimulation (Fig. 1d).

### Neutrophils express the class I PI3K catalytic subunits α, δ and γ

**Fig 2:**
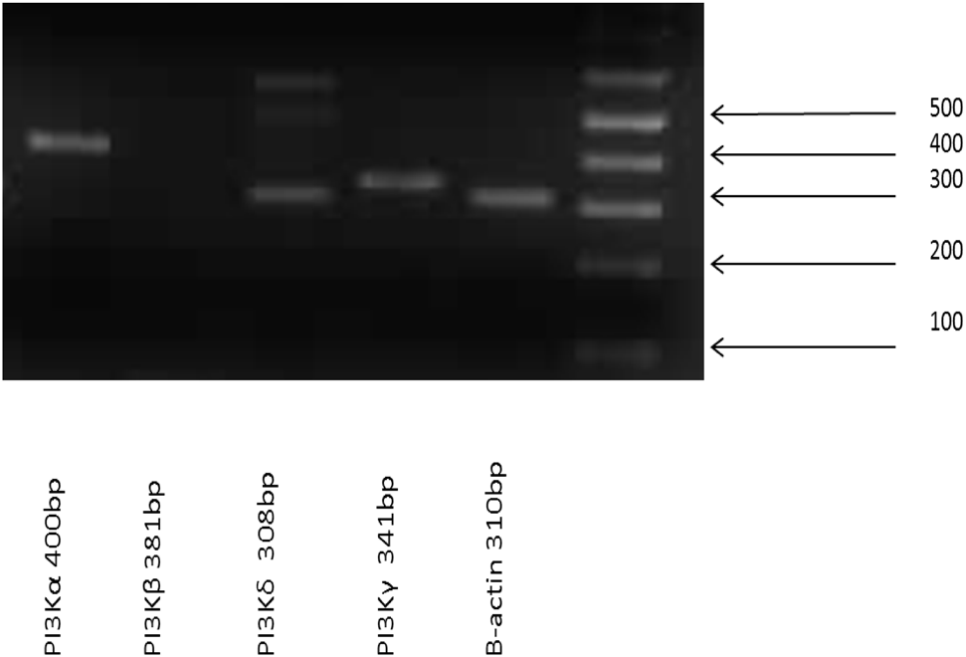
Neutrophils express the class I PI3K catalytic subunits α,δ and γ. RNA was isolated from unstimulated neutrophils. RT-PCR was carried out using primers for each of the four class I PI3-kinase catalytic isoforms and β-actin as a control. n = 1 representative of three experiments. The identity of each of the bands was confirmed by excising the band and sequencing the DNA product.

Primers specific to the class I PI3K catalytic isoforms α, β, δ and γ were designed and RT-PCR was used to determine their expression (Fig. 2). The isoforms α, δ and γ were expressed in all three experiments using different neutrophil donors. The β isoform was detected in one of the three experiments. From the experiment where all four isoforms were expressed the DNA was extracted from the gel and was sent for sequencing which confirmed that each band was specific to the isoform of interest. As the α, δ and γ isoforms were always detected their role in neutrophil migration was further investigated.

### Differential requirements for PI3K

**Fig 3:**
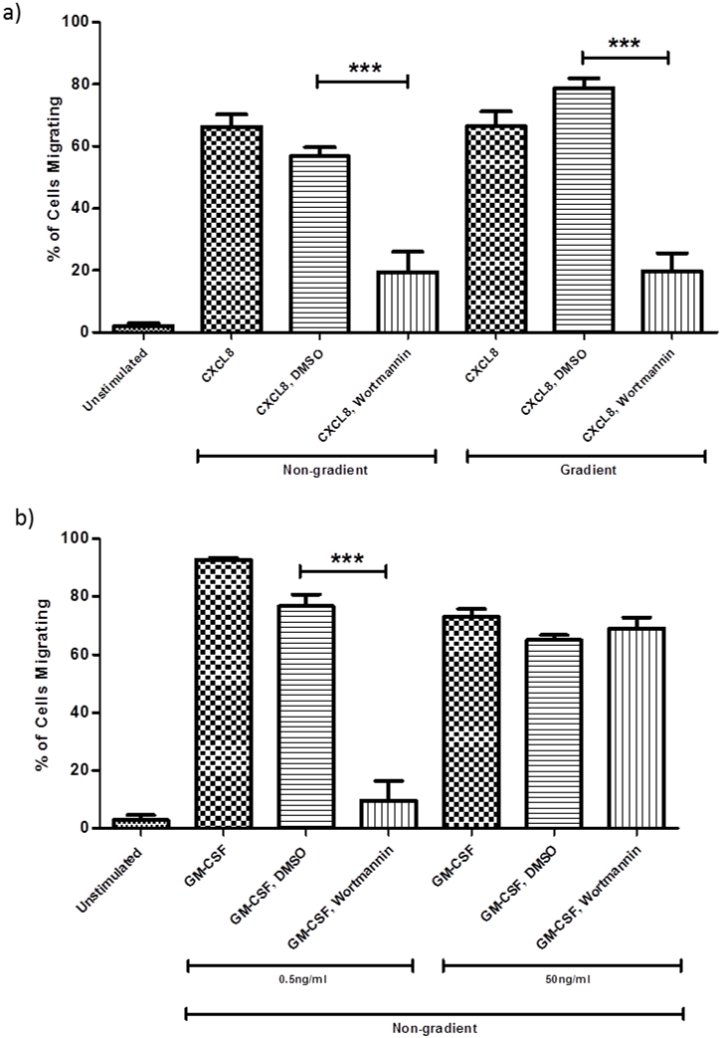
Wortmannin inhibits both CXCL8 and GM-CSF-mediated neutrophil migration. Neutrophils were pre-treated for 30 mins with 50nM wortmannin, and then stimulated with a) CXCL8 (100ng/ml) or b) GM-CSF (0.5ng/ml, 50ng/ml). Results are shown as mean ±SEM (n = 7) except for DMSO controls and 0.5ng/ml GM-CSF where n = 3, ***p<0.001.

PI3K is thought to play an important role in cell migration. The pan-inhibitor wortmannin was used initially in order to determine the importance of PI3K in neutrophil motility in our 3D assay. Treatment of neutrophils with 50nM wortmannin for 30 minutes prior to the addition of chemoattractant significantly inhibited CXCL8-induced migration in both the gradient and non-gradient assays (Fig. 3a). In the non-gradient assay treatment of cells with wortmannin (50nM, 30 min pre-treatment) significantly reduced migration in response to stimulation with 0.5ng/ml GM-CSF but had no significant effect on migration in response to stimulation with 50ng/ml GM-CSF (Fig. 3b). The vehicle control for wortmannin (0.3% DMSO) had no significant effect on neutrophil migration induced by either CXCL8 or GM-CSF (Fig. 3a, b). These data highlight that PI3K is necessary for neutrophil migration in 3D gels in response to stimulation with CXCL8 and a sub-optimal concentration of GM-CSF.

### The role of PI3Kγ in neutrophil migration

**Fig 4:**
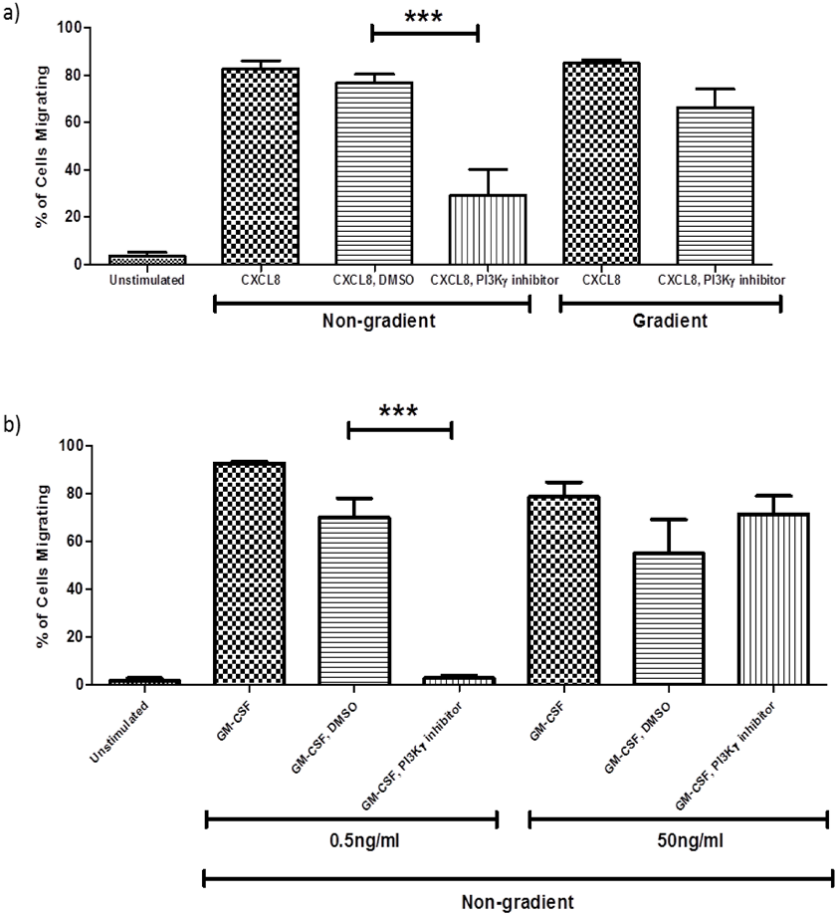
An antagonist of PI3Kγ inhibits CXCL8 mediated chemokinetic but not chemotactic migration and GM-CSF mediated chemokinetic migration at a sub-optimal concentration of GM-CSF. Neutrophils were treated with 10μM PI3Kγ selective inhibitor for 30 mins prior to the addition of CXCL8 (100ng/ml, a) or GM-CSF (0.5ng/ml, 50ng/ml, b). Data shown are mean ±SEM (n = 3) except for non gradient CXCL8 where n = 4 and GM-CSF where n = 6 ***p<0.001.

Neutrophils were pre-treated with the PI3Kγ selective inhibitor AS-605240 (10μM), for 30 minutes prior to addition of chemoattractant. In the non-gradient assay CXCL8-induced neutrophil migration was significantly inhibited by AS-605240. In contrast AS-605240 had no effect on CXCL8-induced migration in the gradient assay (Fig. 4a). In GM-CSF-induced neutrophil migration pre-treatment with the PI3Kγ selective inhibitor completely abolished migration when cells were stimulated with 0.5ng/ml GM-CSF but had no significant effect on migration when cells were stimulated with 50ng/ml GM-CSF (Fig. 4b). The vehicle control for the PI3Kγ selective inhibitor (3% DMSO) had no significant effect on neutrophil migration induced by either CXCL8 or GM-CSF (Fig. 4a, b).

### The role of PI3Kδ in neutrophil migration

**Fig 5:**
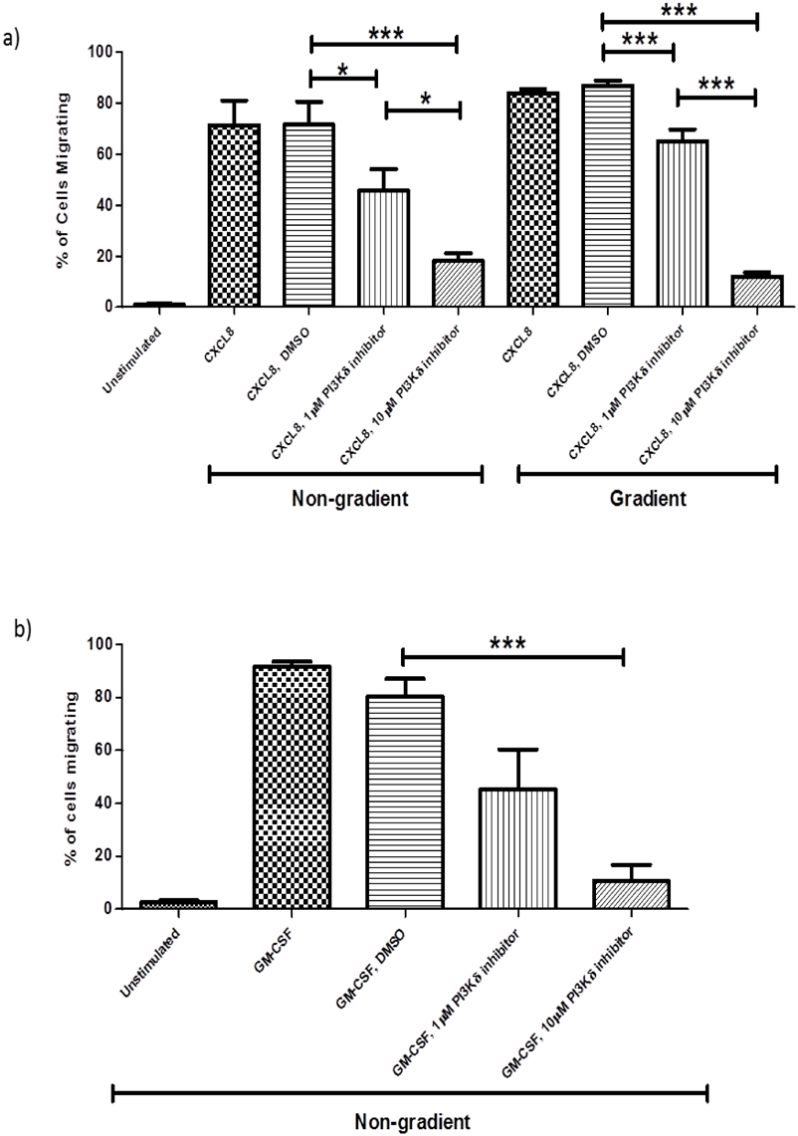
An antagonist of PI3Kδ inhibits both chemotactic and chemokinetic migration mediated by CXCL8 and GM-CSF-. Neutrophils were treated with 1μM and 10μM of the PI3Kδ selective inhibitor PIK-274 for 30 mins prior to the addition of CXCL8 (100ng/ml, a) or 0.5ng/ml GM-CSF (b). Data shown are mean ±SEM (n = 3) except for gradient DMSO and 1μM of PIK-274 where n = 8. *p<0.05, ***p<0.001.

**Fig 6:**
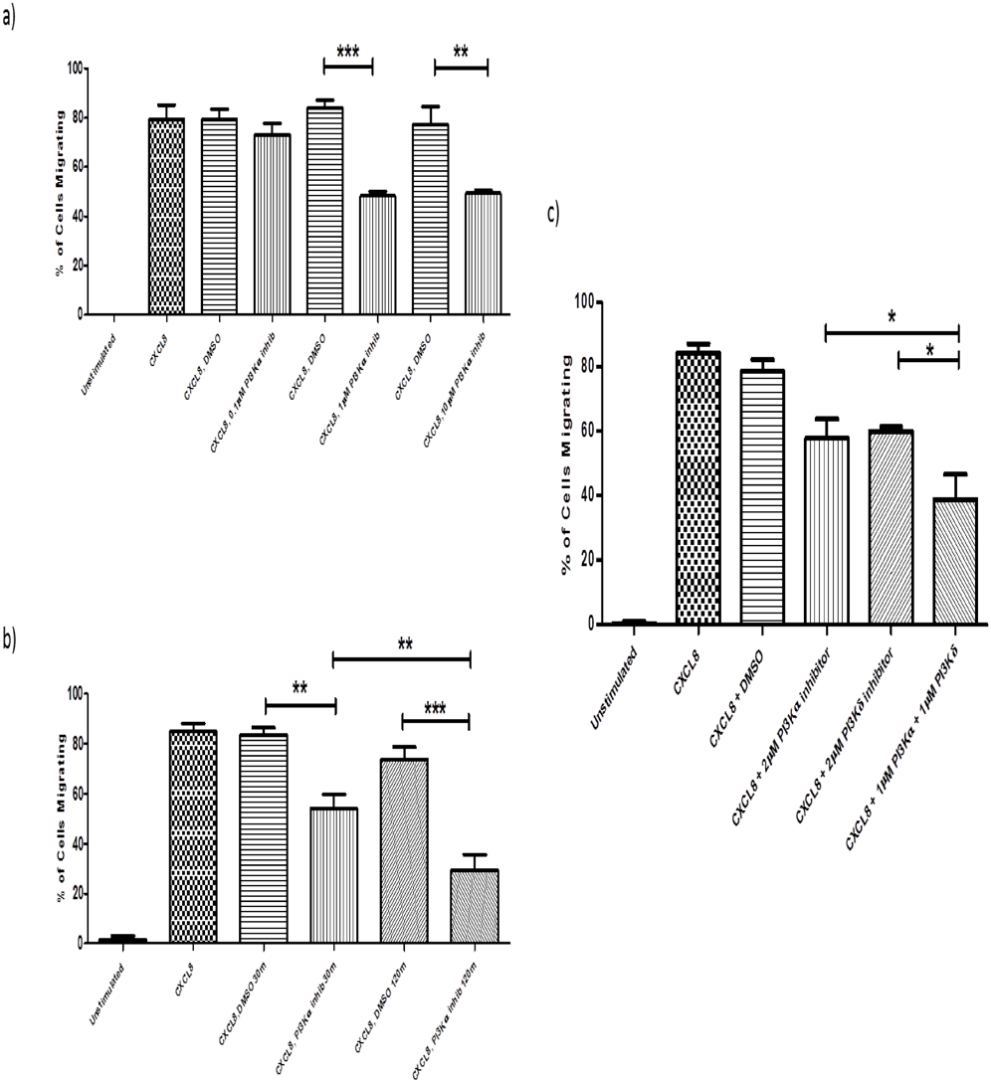
An antagonist of PI3Kα inhibits CXCL8 induced chemotactic migration in a dose and time dependent manner. a) Neutrophils were pre-treated for 30 mins with varying concentrations (from 0.1μM to 10μM) of the PI3Kα selective inhibitor PIK75, and then stimulated with 100ng/ml CXCL8 in the gradient assay. b) Neutrophils were pre-treated with 1μM of PIK-75 for 30 minutes or 2 hours, prior to construction of the migration gel and the addition of 100ng/ml of CXCL8 in the gradient assay. Results are shown as mean ±SEM; n = 4, except for CXCL8, 1μM and the corresponding DMSO control where n = 11 ***p<0.001, **p<0.01. **Antagonists of PI3Kδ and α have additive effects on inhibition of CXCL8 mediated neutrophil chemotaxis.** Neutrophils were pre-treated for 30 mins with either 2μM of the PI3Kα selective inhibitor, PIK-75, 2μM of the PI3Kδ selective inhibitor, PIK-274 or 1μM of each and then stimulated with 100ng/ml CXCL8. Results are shown as mean ±SEM (n = 4) *p<0.05. (Fig. 6c)

When cells were pre-treated with the PI3Kδ selective inhibitor PIK-294 CXCL8-induced migration in both assays was significantly inhibited (Fig. 5a). The inhibitor was used at two concentrations 1μM and 10μM. Pre-treatment with 1μM inhibited migration to a greater extent in the non-gradient assay than in the gradient assay. Pre-treatment with 10μM inhibited migration to a significantly greater extent than the lower dose in both assays. With GM-CSF-induced migration treatment with the PI3Kδ selective inhibitor at a concentration of 10μM almost abolished migration (Fig. 5b). The vehicle control for the PI3Kδ selective inhibitor (0.1% DMSO) had no significant effect on neutrophil migration induced by either CXCL8 or GM-CSF (Fig. 6a, b). These results indicate that PI3Kγ is necessary for chemokinetic migration whereas PI3Kδ is vital for both chemokinetic and chemotactic migration in response to stimulation with CXCL8 or GM-CSF.

### The role of PI3-kinase α in neutrophil migration

The catalytic isoform PI3Kα has not been previously reported to be involved in controlling neutrophil migration. However, as the α isoform was shown to be expressed in neutrophils we decided to determine if it was involved in migration. A selective inhibitor of PI3Kα, PIK-75, significantly inhibited CXCL8 mediated neutrophil migration in the gradient (chemotactic) assay in a dose dependent manner with maximal inhibition seen with 1μM and 10μm concentration (Fig. 6a). The vehicle control for PI3Kα (0.5%DMSO) had no significant effect on neutrophil migration. In a time course experiment inhibition was seen with 1μM at both 30 minutes and two hours with a more marked effect at the longer time point. No inhibition of migration was seen with the DMSO control (Fig. 6b) In contrast to the gradient (chemotactic) assay in the non-gradient (chemokinetic) assay the PI3Kα selective inhibitor had no effect on either CXCL8-or GM-CSF induced migration (data not shown).

### Treatment of cells with the PI3Kα selective inhibitor and PI3Kδ selective inhibitor in combination

As PI3Kα and δ were both shown to play a role in chemotactic migration the effect of the two inhibitors when added in combination was examined. In the gradient assay neutrophils were pre-treated with the PI3Kα selective inhibitor alone (2μM, 30min pre-treatment), the PI3Kδ selective inhibitor alone (2μM, 30min pre-treatment) or the inhibitors in combination (1μM of each inhibitor, 30min pre-treatment). Both inhibitors as previously demonstrated inhibit CXCL8-induced neutrophil migration, treatment with the inhibitors in combination significantly increased inhibition of neutrophil migration in comparison to the inhibitors alone (Fig. 6c). The vehicle control for the inhibitors (0.5%DMSO) had no significant effect on neutrophil migration. The patterns of inhibition seen with the PI3 kinases inhibitors are summarised in Table 1.

**Table 1:** Summary of the degree of inhibition obtained with the PI3 kinase inhibitors on CXCL-8 and GMCSF induced neutrophil migration.

	Non-Gradient/Chemokinetic				Gradient/Chemotactic			
	Wort	γ	δ	α	Wort	Γ	δ	α
CXCL8	++	++	++	-	++	-	++	++
GMCSF 0.5ng/ml	+++	+++	++	-	ND	ND	ND	ND
GMCSF 50ng/ml	-	-	ND	-	ND	ND	ND	ND

% inhibition

+++ ~100%

++ >50%

- Not significant

ND: Not done

### CXCL8 and GM-CSF stimulate Akt phosphorylation

**Fig 7:**
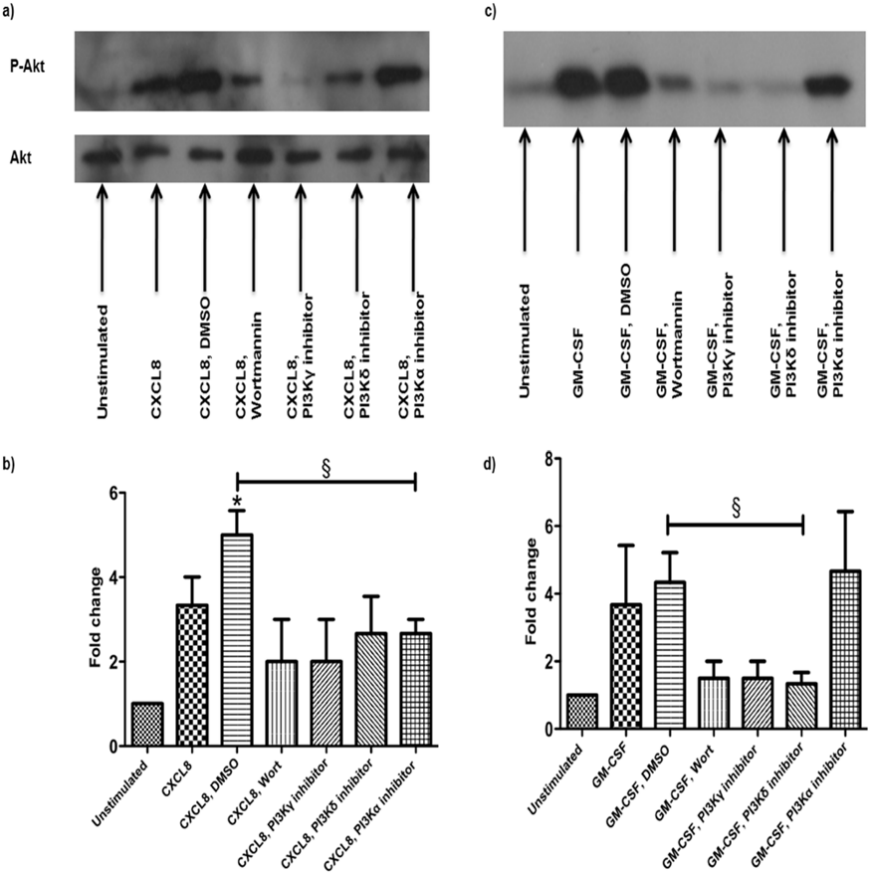
CXCL8 and GM-CSF induce the phosphorylation of Akt. Neutrophils were treated with wortmannin (50nM) or the PI3K (1M), (10M) and (10μM) selective inhibitors for 2 min in combination with 500ng/ml of CXCL8 (a,b) or 50ng/ml GM-CSF (c,d). Western blots were carried out using the antibodies Phosphorylated Akt and total Akt (a,c). Densitometry was performed on the western blot films (b,d). The western blots shown are an example of 1 of the 3 carried out which all demonstrated the same pattern. In Fig. 7b CXCL8+DMSO significantly increased phosphorylation of Akt compared to the unstimulated control (p<0.05) and PIK-75 (PI3kinase α) caused significant inhibition of phosphorylation (p<0.05). In Fig. 7d the PI3kinase δ inhibitor PIK-294 significantly inhibited GM-CSF+DMSO mediated phosphorylation (p<0.05)

To determine the activation of PI3Ks phosphorylation of one of the downstream effectors of the PI3Ks can be examined. Phosphorylation of Akt was therefore examined. Western blotting shows that stimulation with CXCL8 with the DMSO control for the inhibitors (500ng/ml; 2 mins) increased phosphorylation of Akt in comparison to control unstimulated neutrophils (Fig. 7a, b) (p<0.05). The vehicle control for the inhibitors (0.1% DMSO) had no effect on the phosphorylation of Akt in comparison to the CXCL8 stimulated cells. Prior to stimulation with CXCL8, pre-treatment of the cells with the PI3K inhibitors, wortmannin (50nM), PIK-294 (δ, 10μM) and AS-605240 (γ, 10μM) for 2 minutes, caused a reduction in the phosphorylation of Akt (Fig. 7a, b). Pre-treatment with PIK-75 (α, 1μM) for 2 minutes caused a significant reduction in the phosphorylation of Akt compared to stimulation with CXCL8 and DMSO (Fig. 7a, b)(p<0.05). Akt was used as a control and showed bands of equal size indicating that the loading of each well was approximately the same so the effects seen with the Phosphorylated-Akt are due to differences in the stimulation and activation not in the quantity of cells loaded (Fig. 7a).

Stimulation of neutrophils with GM-CSF (50ng/ml; 2 mins) induced the phosphorylation of Akt (Fig. 7c, d). The vehicle control for the inhibitors (0.1% DMSO) had no effect on the phosphorylation of Akt (Fig. 7c, d). Pre-treatment of cells prior to stimulation with GM-CSF and the DMSO control with the PI3-Kinase inhibitors wortmannin (50nM), PIK-294 (δ, 10μM) and AS-605240 (γ, 10μM) for 2 minutes, reduced the phosphorylation of Akt (p<0.05 for inhibition of PI3kinase delta) however pre-treatment with PIK-75 (α, 1μM) for 2 minutes, had no effect on the phosphorylation of Akt, compared to the vehicle control (Fig. 7c, d).

## Discussion

Neutrophils are normally not abundant in healthy tissue, however during periods of infection and inflammation neutrophil numbers escalate as they leave the blood to migrate to these sites. This is essential for host defence against infection however in some conditions including respiratory diseases including chronic obstructive pulmonary disease, cystic fibrosis and asthma the presence of neutrophils within the tissue can be detrimental leading to tissue damage [23–25].

This study investigated neutrophil migration in a 3D collagen environment designed to mimic the tissue environment of particularly the lung as the collagen accumulation is increased in the asthmatic lung [20, 21, 26]. This has highlighted clear differences in the control of migration in chemokinesis and chemotaxis in relation to signalling via PI3K. The neutrophils for this study were obtained from healthy volunteers in order to determine the normal pattern of migration prior to investigating the migration of cells from diseased individuals. We used CXCL8 as a physiologically relevant and reliable neutrophil chemoattractant that signals through G protein linked seven membrane chemoattractant receptors. fMLP gave a similar pattern of migration to CXCL8 in both the gradient and non-gradient gel (data not shown). We used GMCSF as a known priming and survival agent for neutrophils that was likely to be present at sites of neutrophilic inflammation. GMCSF signals through a tyrosine kinase receptor that mediates chemokinesis, but not chemotaxis. We considered it likely both these mediators would be present together in inflamed tissue and wished to investigate how neutrophils would integrate the responses through each pathway.

Unlike unstimulated neutrophils, which were unable to move, stimulation with both CXCL8 and GM-CSF evoked a high proportion of cell migration. This contrasted with eosinophils, which migrated well to GM-CSF but only to a limited extent to chemotactic stimulation (eotaxin and C5a) in 3D gels [21]. The non-gradient and gradient stimulated respectively chemokinesis and chemotaxis in response to stimulation with CXCL8. In contrast in both the non-gradient and gradient assays migration in response to GM-CSF was chemokinetic. As the non-gradient and gradient migration patterns were essentially the same, we only used the non-gradient assay for subsequent experiments with GM-CSF.

The class I PI3K catalytic subunits p110α and p110β are ubiquitously expressed, whereas in contrast the p110δ and p110γ isoforms are restricted to leucocytes in their expression [16, 27]. These studies and the PCR data we presented show that all four class I PI3K isoforms can be expressed by neutrophils. We saw an additional band for PI3kinase delta which was larger than the predicted size. The identity of this band requires further investigation. A role for PI3Ks in neutrophil migration primarily using a 2D migration system has been previously demonstrated [4, 17, 28–31]. Our data using wortmannin confirmed this involvement. As when the cells were stimulated with CXCL8 or a suboptimal concentration of GM-CSF migration was almost completely abolished. Migration was not affected when the cells were stimulated with the optimal concentration of GM-CSF possibly because this activated the cells to such an extent that the concentration of the inhibitor was not sufficient to block migration although good inhibition of GM-CSF stimulated Akt phosphorylation by wortmannin was seen by western blotting. An alternative explanation is that the higher dose of GM-CSF activated a migration pathway that was not dependent on PI3 kinase phosphorylation of Akt.

A role for the PI3Kδ isoform in migration has been previously described using the under agarose assay [15]. These authors demonstrated that pre-treatment of neutrophils with the PI3Kδ selective inhibitor, IC87114 inhibited chemotaxis, but had no effect on chemokinesis. It was therefore concluded that PI3Kδ was responsible for the directional orientation of neutrophils during migration [15]. Our results confirm the involvement of PI3Kδ in chemotaxis as pre-treatment of the cells with the PI3Kδ selective inhibitor PIK-294 significantly reduced migration in response to stimulation with CXCL8. However in contrast to the above study our results also showed that blockage of PI3Kδ inhibited both CXCL8- and GM-CSF-induced chemokinesis. The neutrophils were pre-treated with two concentrations of PIK-294 and interestingly the lower concentration of inhibitor caused a greater reduction in CXCL8-induced chemokinesis compared to chemotaxis, the higher concentration caused approximately the same reduction in both chemokinesis and chemotaxis. The PI3Kδ inhibitor also antagonises PI3Kγ at a 10 fold higher concentration so we cannot therefore exclude the possibility that some of the inhibition could be due to cross-over inhibition of the γ isoform. However we feel this is unlikely considering the high dose of the PI3Kγ inhibitor required to achieve inhibition of chemokinesis.

Inhibitor and knockout experiments with both human and mouse neutrophils have shown that migration is dependent on PI3Kγ [17, 32–35]. Rather than being involved in both chemokinesis and chemotaxis it is thought that PI3Kγ may only be important for chemokinesis as Ferguson [17] reported that pre-treatment with a PI3Kγ selective inhibitor significantly reduced the number of cells migrating when stimulated with fMLP in a Dunn chamber but had no effect on the migratory index, speed or direction. This lead to the conclusion that inhibition of PI3Kγ results in impaired chemokinesis but not the navigation of cells once they are moving [17]. Our results support this as PI3Kγ inhibitor AS-605240 significantly reduced chemokinesis in the non-gradient assay in response to stimulation with CXCL8. In contrast chemotaxis in the gradient assay was unaffected by pre-treatment with AS-605240. Although migration induced by 50ng/ml GM-CSF in the non-gradient assay was not inhibited by AS-605240, when the cells were stimulated with 0.5ng/ml GM-CSF, pre-treatment with AS-605240 abolished neutrophil chemokinesis. The difference between the inhibition seen with the suboptimal and optimal concentration of GM-CSF was presumably due to the higher concentration of GM-CSF activating the cell to such an extent that the concentration of the inhibitor was not enough to completely prevent phosphorylation of the kinase although some inhibition was seen by western blotting (Fig. 7c). As noted above however it is possible high doses of GM-CSF activate alternative signal transduction pathways. The failure of PI3 kinase inhibitors to prevent neutrophil chemokinesis to high concentrations of GM-CSF is reminiscent of the findings by Ferguson et al [17] that LPS stimulated neutrophil migration was PI3 kinase independent.

This is the first study to show the involvement of the PI3Kα isoform in human neutrophil chemotaxis in response to stimulation with CXCL8. Migration was not completely abolished therefore PI3Kα in addition to one or more of the other isoforms may be vital for chemotaxis. When neutrophils were pre-treated with a combination of both PI3Kα and δ inhibitors, chemotaxis in response to CXCL8 was further reduced highlighting an important combined role of the two isoforms for controlling neutrophil chemotaxis. This also suggests that the two isoforms are working through different pathways. The PI3Kα inhibitor does not have any effect on the δ isoform (see Table 2). It does inhibit the γ isoform with a 13 fold higher IC50 however the γ inhibitor AS-605240 had no effect on gradient migration demonstrating that PIK-75 is indeed mediating its effects by inhibiting the α isoform.

**Table 2:** IC50s of the PI3 kinase isoform inhibitors.

	α	Γ	δ
	PIK-75	AS-605240	PIK-294
alpha (IC50)	**5.8 nM**	60nM	10μM
gamma (IC50)	76 nM	**8nM**	160nM
delta (IC50)	510 nM	300nM	**10nM**

As Akt lies downstream of PI3Ks and may mediate some of the actions of PI3Ks, Akt phosphorylation was examined. CXCL8 has been shown previously to induce Akt phosphorylation, which can be reduced by pre-treatment with wortmannin [30, 36]. In our experiments as expected CXCL8 induced Akt phosphorylation and this was reduced by pre-treatment with wortmannin and the PI3Kδ and γ selective inhibitors, however the PI3Kα selective inhibitor had no effect on the level of Akt phosphorylation. As with CXCL8, treatment with GM-CSF-induced Akt phosphorylation this was reduced by pre-treatment with wortmannin and the PI3Kδ and γ selective inhibitors. The PI3Kα selective inhibitor had no effect on Akt phosphorylation induced by stimulation with GM-CSF. Therefore while Akt may be involved in the migrating activity of PI3δ and γ this does not appear to be the case for alpha. However one caveat is that if we had used a longer pre-incubation time it is possible we would have seen great inhibition of Akt phosphorylation. The role of Akt in neutrophil migration has recently been explored; a deletion in Akt2 was shown to impair neutrophil migration [37]. Akt2 could therefore be an important component in controlling neutrophil migration.

The specificity of pharmacological inhibitors can always be challenged and ideally our observations with pharmacological inhibitors should be confirmed with molecular approaches to knock down the relevant gene and protein. Gene deletion in an animal model is not within the scope of this paper although as noted above such studies have supported our findings using the pharmacological inhibitor of the gamma subunit [34]. Gene deletion of the alpha unit was shown to be embryonically lethal and we could find no papers on gene deletion of the delta subunit [38]. As far as we are aware siRNA approaches to reducing expression of the PI3 kinase isoforms to investigate migration are not possible in freshly isolated human blood neutrophils due to their short life span and low biosynthetic capacity.

The signal transduction events controlling neutrophil migration are complex and remain incompletely understood. As shown by our data and by others [17], PI(3)Kγdependent phosphorylation of PtdIns(3,4)P2 to PtdIns(3,4)P3 (PIP2 to PIP3) is necessary for chemokinesis but does not appear to be involved in directional migration although the surface on which migration occurs is important as PI(3)Kγ also controls adhesion. One advantage of using the collagen gel where migration is relatively adhesion independent is that this removes a confounding factor in interpreting the effects of the isoforms on neutrophil migration. In this paper we have also shown that the delta isoform also plays a part in chemokinesis although whether this is via phosphorylation of PIP2 to PIP3 was not tested. Directed migration (chemotaxis) however appears to be controlled by a combination of the PI(3)Kδ and α isoforms which is likely to be independent of PIP2/3 and also in the case of the α isoform phosphorylation of Akt. Directional migration involves a number of second messengers and how these relate to PI(3)Kδ/α activation is not clear.

Our 3D assay is ideal for distinguishing chemokinesis from chemotaxis allowing dissection of the signal transduction processes involved in each function and the way they are modulated by growth factors compared to chemoattractants. We have made the novel observation that the growth factor GM-CSF can only cause chemokinesis even when presented in a gradient fashion. We have also observed for the first time that the α isoform of PI3K can mediate chemotaxis and confirmed roles for the γ isoform in chemokinesis and the δ isoform in chemotaxis and chemokinesis.
