# Correction: The Role of Phosphoinositide 3-Kinases in Neutrophil Migration in 3D Collagen Gels

**DOI:** 10.1371/journal.pone.0134924

**Published:** 2015-07-31

**Authors:** The PLOS ONE Staff

The following information is missing from the Funding section: This paper was supported by the National Institute for Health Research Leicester Respiratory Biomedical Research Unit. The views expressed are those of the author(s) and not necessarily those of the NHS, the NIHR or the Department of Health.

The publisher apologizes for the error.

## References

[1] Martin KJS, Muessel MJ, Pullar CE, Willars GB, Wardlaw AJ (2015) The Role of Phosphoinositide 3-Kinases in Neutrophil Migration in 3D Collagen Gels. PLoS ONE 10(2): e0116250 doi:10.1371/journal.pone.0116250 25659107 10.1371/journal.pone.0116250 PMC4320071

